# Voiceless Bangla vowel recognition using sEMG signal

**DOI:** 10.1186/s40064-016-3170-9

**Published:** 2016-09-09

**Authors:** S. S. Mostafa, M. A. Awal, M. Ahmad, M. A. Rashid

**Affiliations:** 1 Khulna University of Engineering and Technology, Khulna, 9203 Bangladesh; 2 Centre for Clinical Research, The University of Queensland, Brisbane, QLD Australia; 3 Universiti Sultan Zainal Abidin, 21300 Kuala Terengganu, Malaysia

**Keywords:** ANN, Bangla vowel, Classification, Feature selection, sEMG, Wavelet transform

## Abstract

Some people cannot produce sound although their facial muscles work properly due to having problem in their vocal cords. Therefore, recognition of alphabets as well as sentences uttered by these voiceless people is a complex task. This paper proposes a novel method to solve this problem using non-invasive surface Electromyogram (sEMG). Firstly, eleven Bangla vowels are pronounced and sEMG signals are recorded at the same time. Different features are extracted and mRMR feature selection algorithm is then applied to select prominent feature subset from the large feature vector. After that, these prominent features subset is applied in the Artificial Neural Network for vowel classification. This novel Bangla vowel classification method can offer a significant contribution in voice synthesis as well as in speech communication. The result of this experiment shows an overall accuracy of 82.3 % with fewer features compared to other studies in different languages.

## Background

Language is a powerful tool for self-expression and communication among humans. Human language is unique compared to other living creatures of the universe in terms of grammatical and semantic categories (Hockett 1960; Deacon 1997) and the property of recursion (Hauser et al. 2002). The speech production process involves the lungs which serve as an air reservoir and energy, and the larynx manipulates pitch, volume and houses vocal cords. However, the larynx is used to produce sound. Usually, during normal speech, vocal cords in the larynx vibrate and sound is produced just like a musical instrument.

Bengali or Bangla comes from Indo-Aryan language and became the seventh mostly spoken language (Summary by Language Size 2013). It is spoken by 193 million people around the world (Summary by Language Size 2013). Although some researches have been done in Bangla alphabet recognition, the most of them are in written or acoustic signal; such as, direction code feature based and hidden Markov model based. Hidden Markov Models (HMM) based recognition scheme are used to detect online Bangla handwritten basic characters (Bhattacharya et al. 2007; Parui et al. 2008). HMM based classifier and a nearest-neighbor classifier based on Dynamic Time Warping (DTW) are studied in (Mondal et al. 2009). Bangla handwritten cursive word recognition is presented in (Bhattacharya et al. 2008). Fuzzy logic is also used to classify the Bangla vowels (Kamal et al. 2008).

**Fig. 1:**
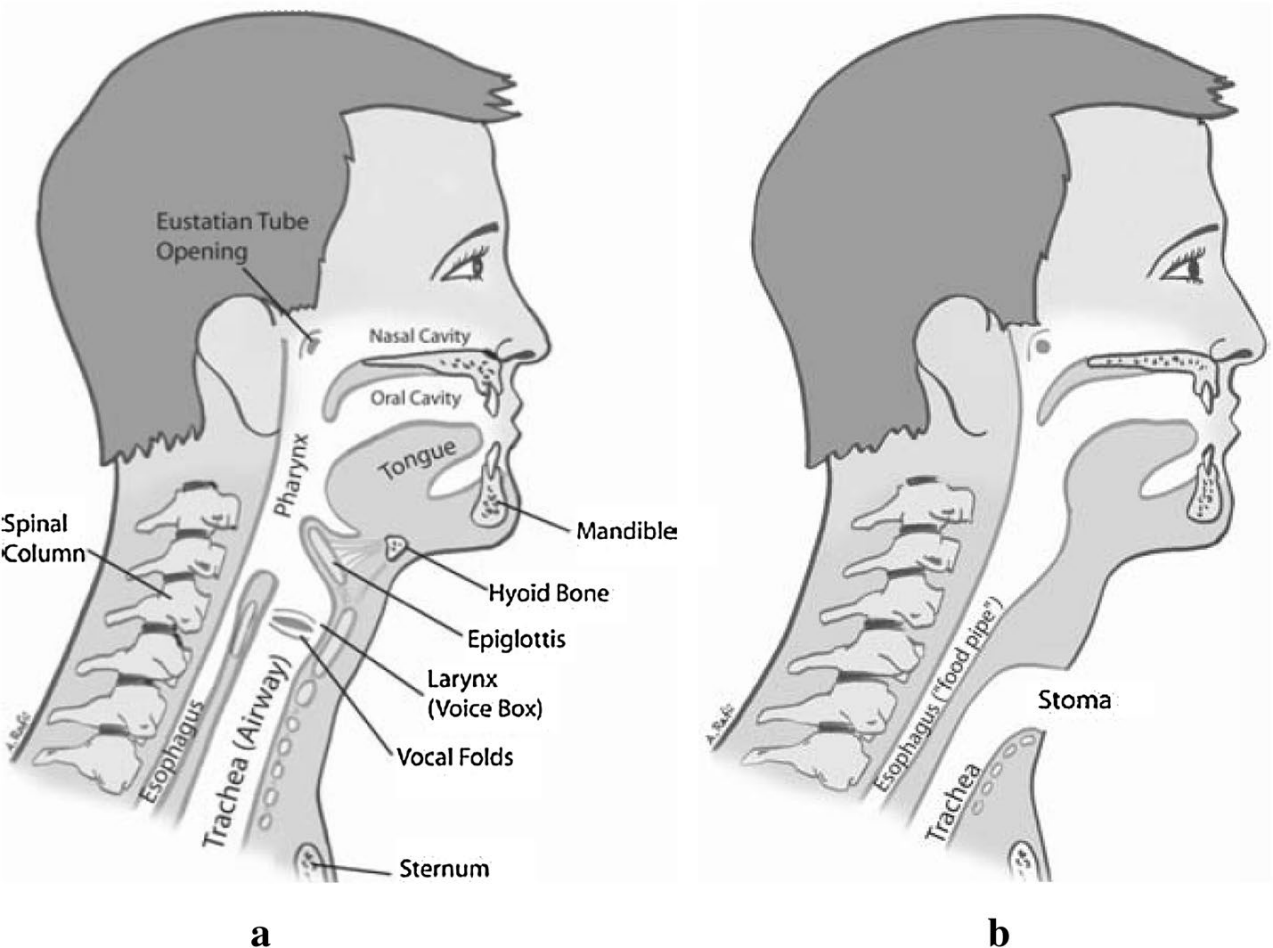
Anatomy of **a** a normal person (with larynx) and **b** a laryngectomy patient

In addition to that, limited robustness in the presence of ambient noise is one of the main drawbacks of traditional speech recognition (Betts et al. 2006). This problem can be solved by using visual sensing technique. However, video based technique (Shanableh et al. 2007; Asadpour et al. 2006) is expensive in terms of computation, sensitive to lighting and need a fix clear view to user lips and mouth (Arjunan et al. 2006). In connection with this discussion, another point can be stated that the people suffered from larynx-cancer are unable to produce speech is growing. For example, it is estimated that 12,720 people (10,110 men and 2610 women) in the USA were diagnosed with larynx-cancer in 2010 (Howlader et al. 2011; Maddox and Davies 2012). It is indisputable that vocal cords play a vital role in the speech production process. Unfortunately, people suffering from the side effect of laryngectomy surgeries or vocal cord damage are unable to produce speech. Laryngectomy patients have their windpipe (trachea) separated from their mouth and food pipe (esophagus) during the operation. They can no longer force air from their lungs through their mouth to speak because their larynx (voice box) is removed. After laryngectomy they will never produce their normal voice sounds. So they need to learn new ways of communicating. Some of the different ways of communication are: using of esophageal voice, an artificial larynx includes esophageal speech (SE), tracheoesophageal (TE) speech and the use of an Electrolarynx (EL). SE speech is difficult to acquire and requires lengthy training because the patient heave to learn swallow and release air in special manner (Gates and Hearne 1982). TE speech is more fluent. However, it needs a surgical procedure and few patients choose to do it due to anatomical or personal reasons (Chenausky and MacAuslan 2000). EL is an external hand-held device which creates buzzing signal. The problem of EL is lack of control over pitch, loudness and onset/offset of sound, robotic and non-human sound quality, reduced intelligibility, and the inconvenience (Meltzner et al. 2003; Shing et al. 2016). Non-acoustic communication systems that use surface electromyogram can solve some limitation of speech commutation (Kumar and Mital 1996). The pitch problem in EL speech for Cantonese is solved using EMG (Shing et al. 2016). Relationship between EMG and nasal vowels for Portuguese is established by real-time magnetic resonance imaging (Freitas et al. 2015). German and English vowel is also recognized by root mean square signal and ANN (Arjunan et al. 2006, 2007). Some researchers used EMG from non-speech muscle to identify English vowels for children (Niu et al. 2014). Myoelectric signal produced in the facial muscles during speech is used to classify (Zhou et al. 2009). Recognizing words from isolated sEMG signals was proposed in Lee (2008), Colby et al. (2009), Wand and Schultz (2010). The system can work under acoustically harsh environment (Chan et al. 2002; Betts and Jorgensen 2005). EMG based speech recognition is used in Arabic (Fraiwan et al. 2011) English (Naik and Kumar et al. 2010). Motivated by these, a voiceless Bangla vowels classification is proposed in this paper using sEMG signal. For classification purpose the property of facial muscle contraction using non-invasive sEMG was used. The time domain sEMG signal is transformed into frequency domain using fast Fourier transform (FFT) as well as time–frequency domain using wavelet transform (WT), and different features are extracted from these time domains signal, FFT signal, and WT signals. Minimum Redundancy Maximum Relevance (mRMR) feature selection algorithm was applied to select prominent features and finally ANN was used for multiclass classification. In the proposed method of Bangla vowels recognition using EMG signals, normal subjects who can produce sound were utilized. The idea is that though laryngectomy patients lose their voice, the way of moving their facial muscle while uttering any letter, word or sentence remains the same as the normal people. Figure 1 shows the anatomy of larynx of a normal person and a laryngectomy patient. Both of their facial muscle structure is same, so the muscle movements do not differ with each other while talking.

This paper is organized as follows: section “Background” describes the background problems and the literature review with proper references as well as our proposed method. The detailed methodologies have been described in section “Proposed methods”, which includes the brief introduction to Bangla vowels, facial electromyography for speech recognition, recording of facial EMG and finally feature extraction and classification method. Section “Results” describes the results and finally the paper concludes in section “Conclusions”.

## Proposed methods

**Fig. 2:**
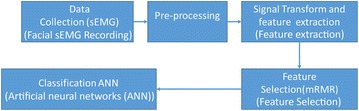
General pipeline of voiceless Bangla vowel classification

The voiceless Bangla vowels classification process started with data collection, then it is pre-processed for de-noising and removing DC components. Then the feature extraction and feature selection algorothm have been appied, and finally classifaction was done using the selected features. Figure 2 shows the flow diagram of the propsed methodolgy.

### Bangla vowels

Bangla script has 50 alphabets that include 39 consonants and 11 vowels. The vowels are called shôrobôrno (স্বরবর্ণ) “vowel letter” in Bangla. However, these vowels may contain 6 or 7 core vowels and two other vowel diphthongs. Compared to consonants vowels are difficult to define because the tongue typically never touches another organ and the shape of the mouth remains constant (Ganesh et al. 2010). The core vowel sound like/æ/has no standard character in the Bangla script. That is why it is not considered in this work. In this paper, we have used 11 vowel letters described in Table 1 (Bengali alphabet 2013).

**Table 1:** Bengali vowel letter chart

Numerical	Alphabetical full form	Name of full form	IPA transcription	Numerical	Alphabetical full form	Name of full form	IPA transcription
1	অ	shôro ô (shôre ô) “vowel ô”	/ɔ/and/o/	6	ঊ	dirgho u “long u”	/u/
2	আ	shôro a (shôre a) “vowel a”	/a/	7	ঋ	ri	/ri/
3	ই	hrôshsho i (hrôshsho i) “short i”	/i/	8	এ	e	/e/and/æ/
4	ঈ	dirgho i “long i”	/i/	9	ঐ	oi	/oj/
5	উ	hrôshsho u (rôshsho u) “short u”	/u/	10	ও	o	/o/
				11	ঔ	ou	/ow/

### Facial electromyography for speech recognition

Human body is treated electrically neutral due to the same number of positive and negative charges. The nerve cell membrane is polarized in the resting state. When a neuron is stimulated the muscle fiber depolarizes as the signal spreads along the surface and muscle fiber contraction happens. This depolarization, along with the movement of ions, makes an electric field near the muscle fiber. An EMG signal is the train of Motor Unit Action Potential (MUAP) showing the muscle response to neural stimulation (Reaz et al. 2006). In case of speech delivery, EMG signals are generated in the facial muscles by opening or closing lips, mouth and jaw as well. Consequently, EMG signals also appear in the extrinsic muscles of the tongue. The number of muscles involved in speech production is very high (Tuller et al. 1981) and therefore, high sensitive EMG device should be used to collect the facial EMG signal.

### Facial sEMG recording

**Fig. 3:**
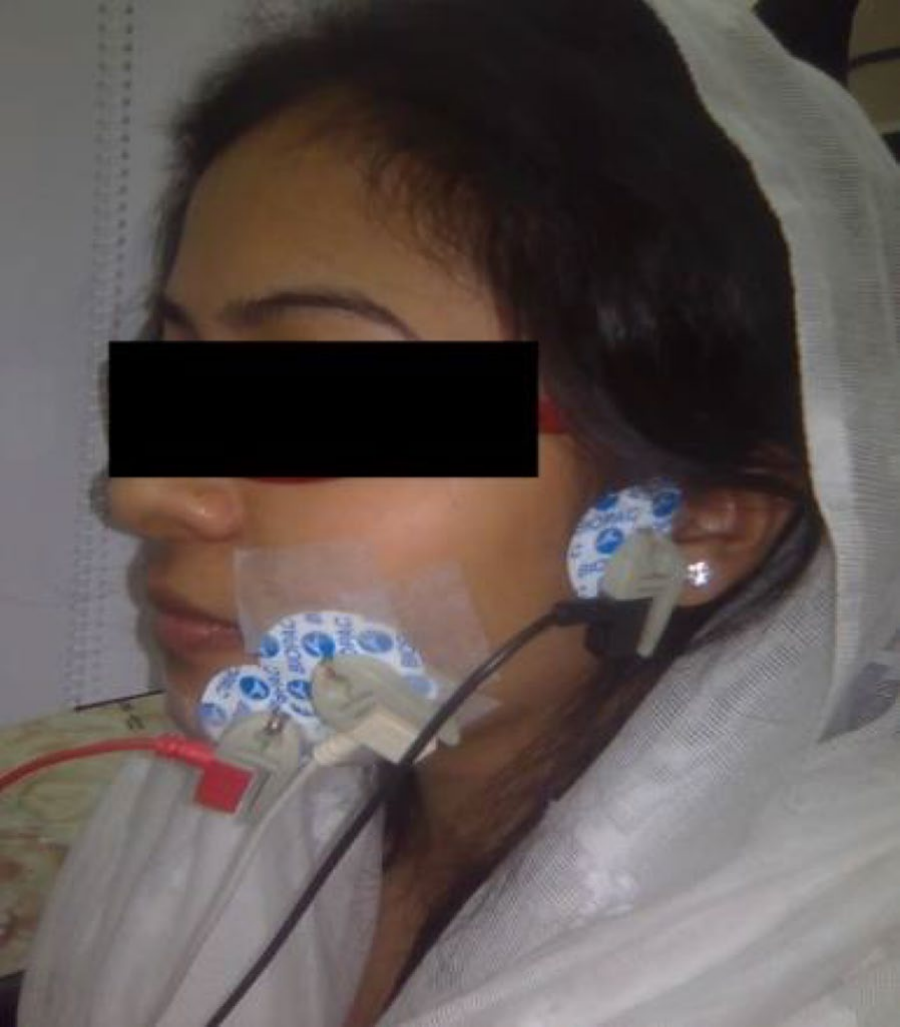
Facial EMG recording of a subject

Fully shielded BSL-SS2LB cable assembly permits high-resolution recording of bio-potentials (sEMG) using disposable vinyl electrodes (EL 503). BIOPAC MP36 data acquisition hardware with a sampling rate of 1000 Hz is used to record and condition electrical signals from the muscle via BSL-SS2LB (channel 3). We used BIOPAC gel 101, formulated with 0.5 % saline in a neutral base to ensure better conductivity between muscle and the electrode. Three sEMG electrodes were placed above the center of the muscle using adhesive tape shown in Fig. 3.

**Fig. 4:**
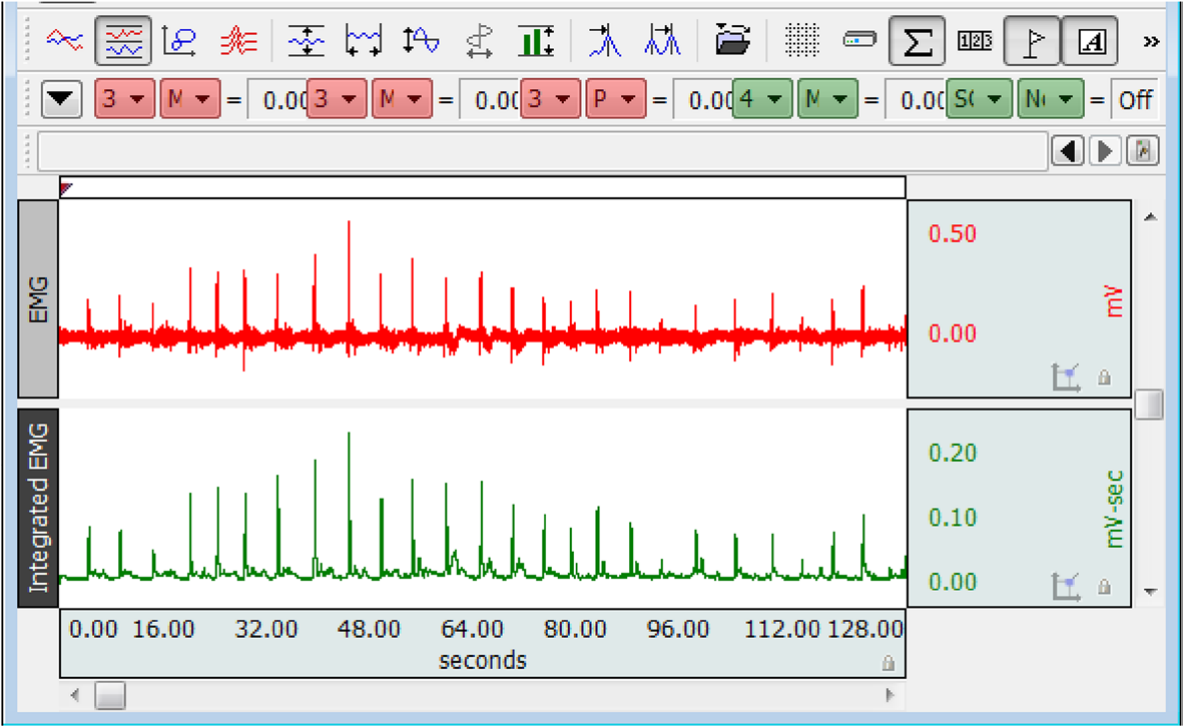
EMG and integrated EMG data extraction

**Fig. 5:**
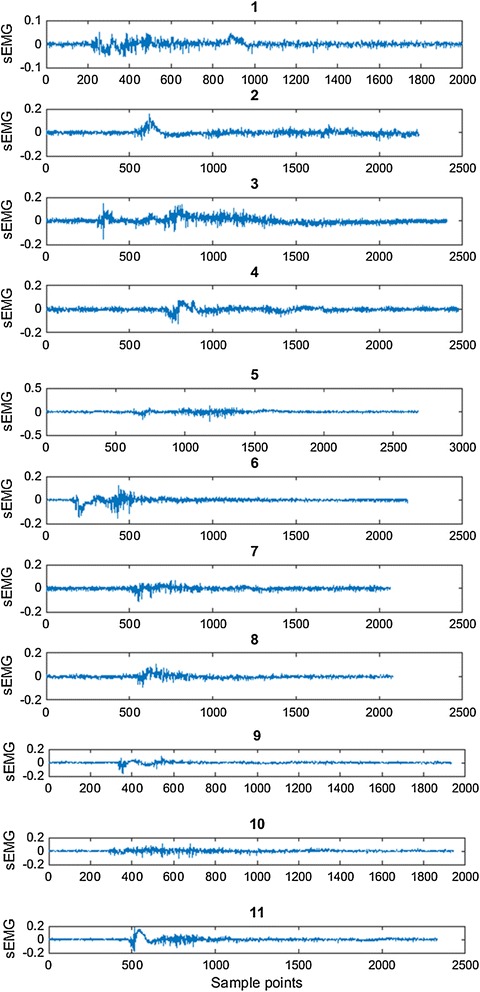
Raw sEMG signal recorded during experiments 1–11 represent the bengali vowel letter from অ to ঔ

Figure 4 shows the extracted EMG and integrated EMG data of a subject. Figure 5 shows the recorded sEMG sample of Bangla vowels letter from অ to ঔ. These types of sEMG were recorded from 8 subjects (2 females, 6 males) mean age 23.5 with standard deviation 0.7559. All the subjects were free from speech impediments or disorders i.e. normal subjects. The subjects participated in this experiment are the university undergraduate students and are native Bangla speakers. No formal training in Bangla phonology and phonetic were provided to the subjects and all subjects were well informed about the whole protocol of the experiment. During the experimental recording session, the subjects were used to sit in front of a computer screen and the EMG sensors were placed on the skin surface of face according to Fig. 3. This study was conducted with approval from the Biomedical Research Ethics Committees of the Khulna University of Engineering and Technology, Khulna, Bangladesh. To reduce the posterior complexity of the system, EMG electrodes were placed on muscles only on one side of the face, since they are symmetric. The final muscles selection and EMG locations are shown in Fig. 3. The black electrode, white electrode and red electrode are placed on masseter muscles, buccinators muscles, and Depressor muscles, respectively.

### Pre-processing

The DC component is removed from the EMG signals by subtracting the signal from its mean value. Then, the signal is band pass filtered in [0.15–450] Hz to filter out the high and low artifacts (such as motion artifacts). This frequency range has a maximum trade-off between suppressing artifacts and retaining the true EMG signal. Next an infinite Impulse Response (IIR) band stop filter (Frequency 50 Hz) was used to filter out electrical noise. The vowels were pounced in discrete sequence in a continuous sEMG recording. So for the classification process each vowel is isolated manually and leveled to extract feature and classification.

### Feature extraction

The features used in this work can be broadly divided into four separate categories: time based, frequency based, entropy based and time–frequency based features. These features are shown useful in different applications and hence adapted in this paper. A brief description of the features are described here and summarized in Table 2.

**Table 2:** Features employed in this study

Domain	Name of the features	Number of features
Time based	Average, maximum, standard deviation, minimum, variance, CoV, skewness, kurtosis, RMS	9
Entropy based	Hjorth mobility, Hjorth complexity, mean of lower envelop, mean of upper envelop, mean diff operator, Higuchi fractal dimension, Higuchi algorithmic entropy, renyi entropy, shannon entropy, Tsallis entropy, Hurst exponential, approximate entropy, sample entropy	13
Frequency based	Spectral flatness, spectral flux, spectral entropy, spectral edge frequency (SEF) 80, spectral edge power (SEF) 80, spectral edge frequency (SEF) 90, spectral edge power (SEF) 90, spectral edge frequency (SEF) 95, spectral edge power (SEF) 95	9
Time–frequency	Symlet 4 wavelet family and decomposition level:3. Entropy (e), variance(V), standard deviation, median (s), average, ration of maximum and min of 4 decomposing s vector’s. Percentage of energy corresponding to the approximation, percentage of energy corresponding to the details	26
–	–	Total 57

1. Time based features: Time based features were calculated from the pre-processed signal directly. Different statistics such as average, maximum, standard deviation etc. of the signal were used as time-based features (see Table 2).
2. Entropy based features: In information theory, entropy is the expected value of the information contained in signal. This paper used different entropies such as Renyi entropy, Shannon entropy and Tsallis entropy etc. as features presented in Table 2.
3. Frequency based features: For frequency based features first the preprocessed signal is transformed into frequency domain and then different frequency domain features such as spectral flatness, spectral flux, spectral entropy etc. are calculated (see Table 2).
4. Time–frequency based features: For Time–frequency based features, first the preprocessed signal is transformed into Time–frequency domain using Discrete Wavelet Transform (DWT). Note that, wavelet is a time-scale transform, can be converted to time- frequency as scale is inversely proportional to frequency. A modified version of Daubechies wavelet families called Symlet wavelet is used in this paper. The rationale is that Daubechies wavelet families are very asymmetric in nature because they are generated by choosing the minimum phase square root (Mallat 2008). It can be shown that filters equivalent to a minimum phase square root concentrate their optimal energy near the initial point of their support (Oppenheim et al. 1999). Therefore, Daubechies wavelets are non-symmetric. To achieve a symmetric or anti-symmetric wavelet the conjugate mirror filter *h*(*n*) must be symmetric or anti-symmetric corresponding to the center of its support. It means that $\hat{h}(\omega )$ has a linear complex phase (Mallat 2008). Daubechies proved that the Haar filter is the only real compactly supported conjugate mirror filter that has a linear phase (Daubechies 1988). Therefore, to obtain more symmetric filter of Daubechies (hence the name “Symlet”) the choice of the square root should be optimized to get nearly the linear phase (Mallat 2008). The resulting wavelets should maintain the minimum support [−*p* + 1, *p*] with *p* vanishing moments like Daubechies wavelets but they are more symmetric. This modified version of Daubechies wavelet is known as Symlet wavelet and has significant contribution in signal and image processing, and can preserve better spectral information (Arivazhagan and Ganesan 2003). In this paper the Symlet wavelet with *p* = 4 vanishing moments (Sym 4) were used.

The DWT composition can be represented by

1 $$f(t) = \sum\limits_{k = - \infty }^{\infty } {C_{N,K} \varphi (2^{ - N} t - k) + \sum\limits_{j = 1}^{\infty } {\sum\limits_{k = 1}^{\infty } {d_{j,k} 2^{{\frac{ - j}{2}}} \psi (2^{ - N} t - k)} } }$$

Here C_N,K_ represents approximate coefficients of level N while *d*_*j*_ (j = 1, 2, …, *N*) represents detailed coefficient or wavelet coefficient at level j. ψ(t) is the wavelet while φ(t) is the scaling function. Now if we relate above equation with filter bank point of view using multi-resolution analysis and filter bank theory, the above equation can be written as (Awal et al. 2011):

2 $$f(t) = \sum {a_{L,k} (t)\phi_{L,k} (t) + \sum\limits_{j = 1}^{L} {\sum\limits_{k \in z} {d_{j} (k)\psi_{j,k} (t)} } }$$

where, *d*_*j*_(*n*) and *c*_*L*_(*n*) can be written as

3 $$d_{j} (n) = \langle f,\psi_{j,n} \rangle = \frac{1}{\sqrt 2 }\sum\limits_{k} {g(2n - k)a_{j - 1} (n)}$$

4 $$c_{L} (n) = \langle f,\varphi_{j,n} \rangle = \frac{1}{\sqrt 2 }\sum\limits_{k} {h(2n - k)a_{L - 1} (n)}$$

The Symlet 4 wavelet was decomposed to a level of three. This is chosen by an experimental search that provides better classification accuracy. Different features were extracted from this wavelet transform and tabulated in Table 2.

### Feature selection

Feature selection is the process of selecting a subset of prominent features for use in classification model construction. Feature selection techniques are able to simplify the models to make them easier to interpret by researchers/users, shorten training times, enhanced generalization by reducing overfitting problem. In this paper, the minimum Redundancy Maximum Relevance (mRMR) feature selection algorithm is used. This feature selection algorithm is based on mutual information (Peng et al. 2005) and shown prominent results in different applications. If x and y are two random variables, their mutual information is defined in terms of their probabilistic density functions:

5 $$I(x;y) = \iint {p(x,y)\log \frac{p(x,y)}{p(x)p(y)}dxdy}$$

Largest dependency on the class registering largest mutual information I(xi;c) within the target class c is calculated individually among selected features $x_{i} \, i = 1,2 \ldots ,S$. The mean value of all mutual information values between individual features x_i_ and class c defined as

6 $$Relevence(S,c) \, D = \frac{1}{\left| S \right|}\sum {I(x_{i} ,c)}$$

MIN-Redundancy selects the features that are mutually maximally dissimilar, which can be expressed in the following form

7 $$Redundancy(S) \, R = \sum\limits_{{x_{i} ,x_{j} \in S}} {I(x_{i} ,x_{j} )}$$

The mRMR ranks features by simultaneously minimizing the redundancy and maximizing the relevance. This operation is implemented by an operator *φ*.

8 $$\hbox{max} \phi (D,R) = D - R$$

### Artificial neural networks (ANN)

Identifying the particular muscles for speech is difficult. Compared to other parts of the body, the Facial structure is more complex and large number of overlaps muscles. Due to unknown aspect of the muscle groups, neural network is used by researchers (Ganesh et al. 2010). Artificial neural networks (ANN’s) having ability of generalization, to learn from experience and to modify itself according to the altering environment makes it unique. Because of these properties, ANN’s is very useful for the classification and discrimination of nondeterministic and extremely disturbed images or signals. ANN is already used in EMG analysis such as force estimation by (Mostafa et al. 2012) or classification (Subasi et al. 2006).

ANN classifier of this paper uses the supervised learning method. Here selected features from the mRMR method are used as inputs(*n*_*i*_) to the network. This is associated with eleven outputs (*n*_*o*_)that is the target. The classifier network consists of two layers of neurons. One hidden layer is created by using hyperbolic tangent sigmoid transfer function. As the input and output neurons are defined by number of inputs and outputs of the system the main difficulty is lies determining number of neurons in the hidden layer, without increasing the unnecessarily complexity. Kolmogorov’s Mapping Neural Network Existence Theorem which is based on the interpretation of the Kolmogorov’s superposition theorem of continuous functions as an ANN (Ciuca and Ware 1997). According to this theorem if input layer consist of *n*_*i*_ inputs where *n*_*i*_ ≥ 2, the numbers of neurons in the hidden layer should be 2*n*_*i*_ + 1 (Gupta, Jin, & Homma 2004). Output layer also uses hyperbolic tangent sigmoid transfer function with eleven neurons. Back propagation algorithm is used to calculate the derivatives of performance with respect to the weight and bias variables. Although this system uses scaled conjugate gradient algorithm based on conjugate directions, it does not carry out a line search in each iteration. The input data is divided in three different sections such as training (70 %), validation (15 %), testing (15 %). The selection process of data is done randomly. In the training section validation of data is used to check the system performance and to prevent overfitting. This overfitting problem is solved by discontinuing the training when the validation error stated to increase or became flat even the training error is in decline. Finally, the test data is used for testing.

## Results

A new method for recognizing the vowels from the Bangla language has been proposed in this paper. The mayoelectric behavior of the facial muscle was used to characterize the vowels using non-invasive sEMG. The time domain, frequency domain, time–frequency domain features were employed for the classification using ANN. At first the feature selection procedure is done by using minimum Redundancy Maximum Relevance algorithm to choose 12 most prominent features from all the features shown in Table 2. They are Spectral Flux, Tsallis entropy, Spectral Entropy, Spectral Edge power (SEF) 80, Spectral Edge power (SEF) 90, wavelet entropy in detailed coefficient at level 1 and level 3 (cd3 and cd1), skewness, min, EA (percentage of energy corresponding to the approximation), average at cd3. In the case of ANN, as 12 input neurons(*n*_*i*_ = 12) is chosen because of 12 features the number of hidden neurons is 25 (2*n*_*i*_ + 1 = 25) (Gupta et al. 2004). The tenfold cross validation can recognize 84.2691 % of the Bangla vowels accurately. This is expected due to many reasons. Firstly, during the pronunciation of vowels, there are high correlation during the starting and ending of the vowels’ pronunciation. Therefore, the classifier sometimes gets confused and lead to misclassification. Feature selection and the classifier may be the other causes that hampered a little bit in the accuracy.

**Fig. 6:**
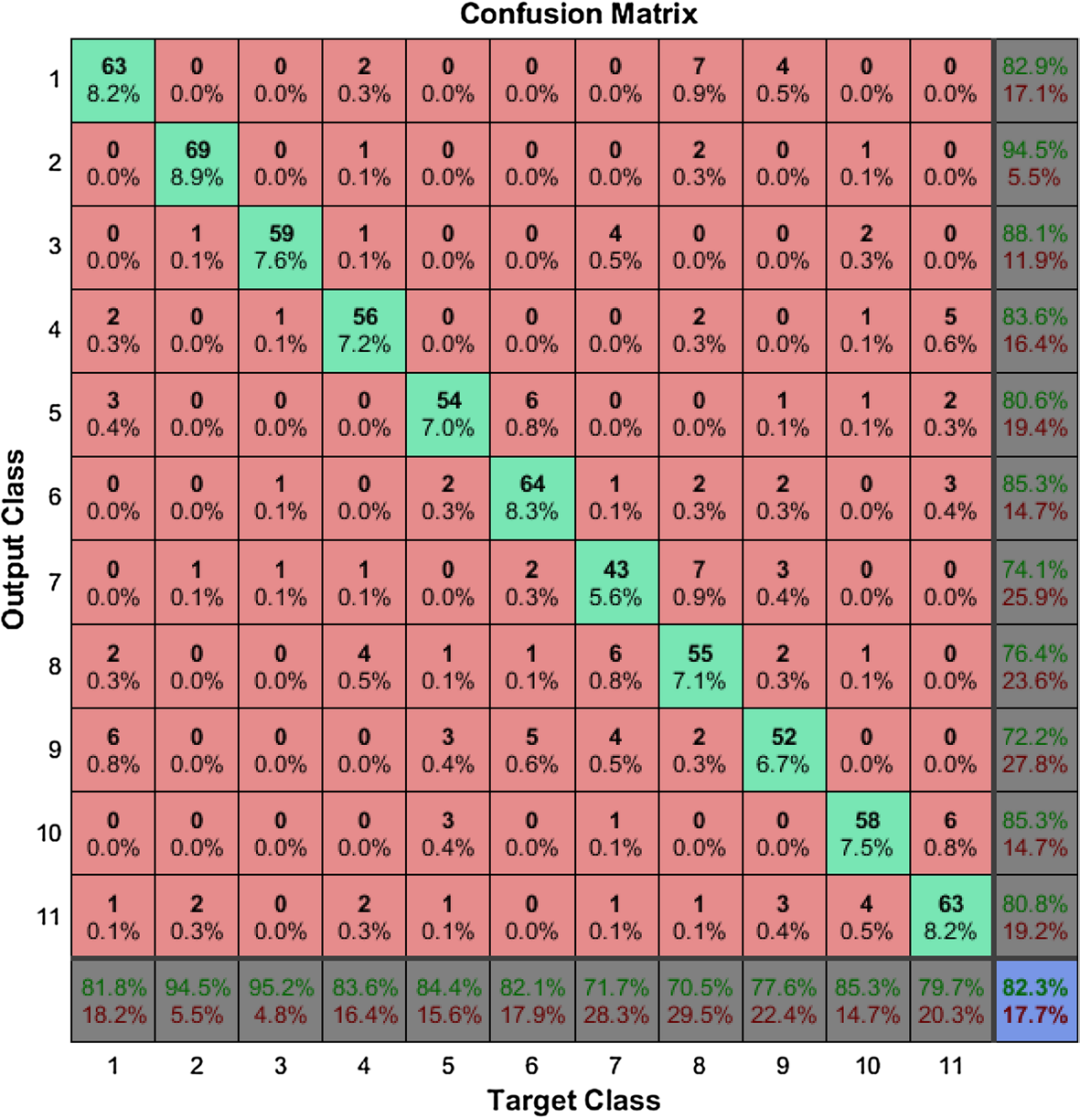
Confusion matrix for the Bangla vowels

For making the confusion matrix, the Bangla letters are represented by numeric symbols, which are shown in Table 1. In the confusion matrix (Fig. 6) which is one of tests of ten cross validations, 1 represents অ, 2 represents আ, and so on. The highest individual accuracy 94.5 % was gained by 2(আ) only two of আ is classified as ঔ, one is ই and another one is ঋ. Eight of eleven calass has more than 80 % indivudal accuracy. ঋ(7), এ (8), ঐ (9)have accuracy between 72 and 75 %. The worst individual classifaction was ঐ (9) which has only 72.2 % correct rate. Most of ঐ (9) error comes from both ঐ (9) is treated অ (1)and অ (1) is treated as ঐ (9)its interesting because the sound অ (1) and ঐ (9) is not similer. Though ই and ঈ sound almost same in Bangla, the ANN classification system does not confuse with them. However, that is not true with উ & ঊ. Two of the উ is classified as ঊ where six of ঊ is classified as উ. The total classification reached 82.3 % which means 17.7 % vowels were classified wrong.

**Fig. 7:**
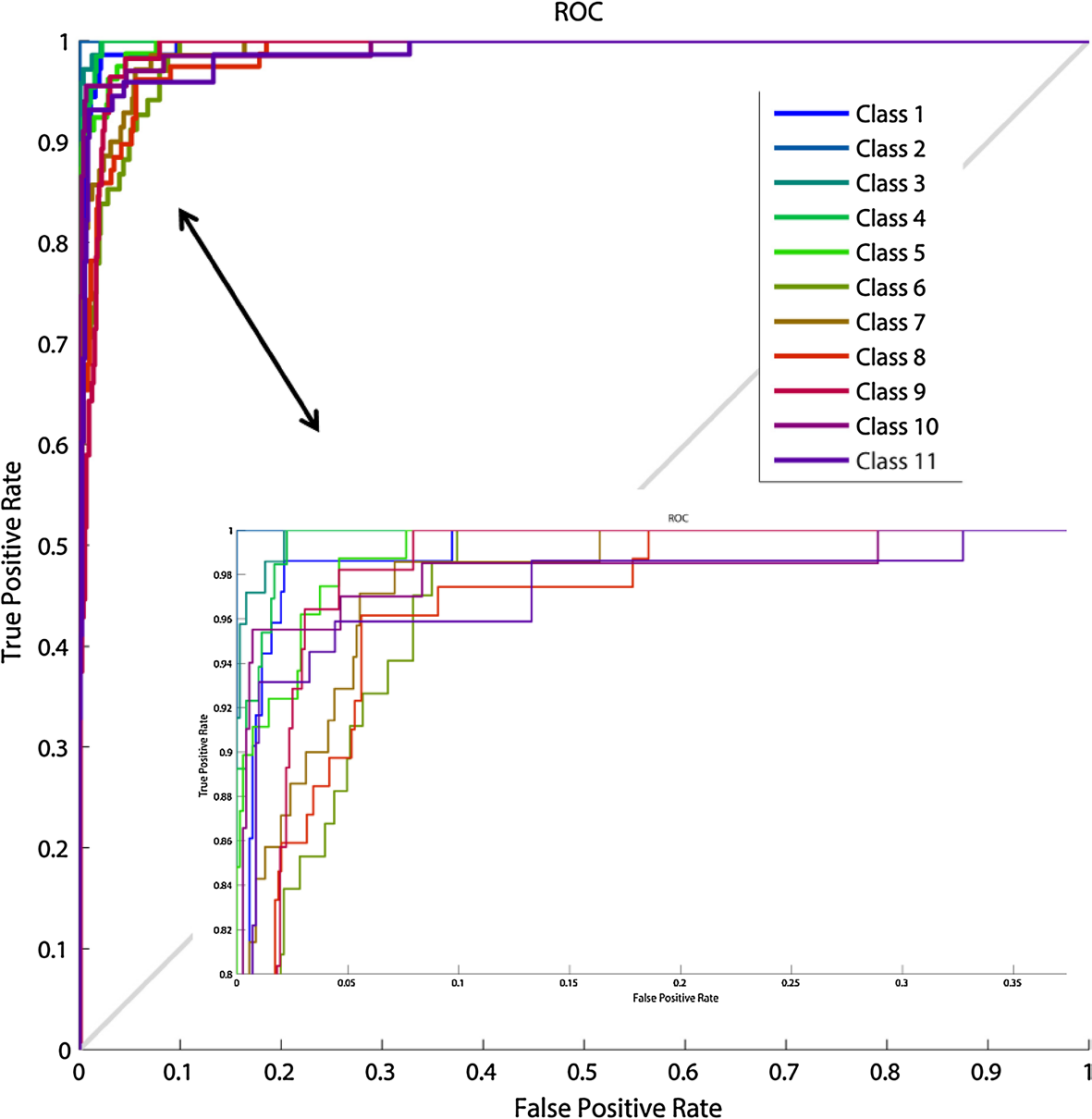
ROC curve for Bangla vowels. The zoomed version of the figure is shown inside the figure

Receiver operating characteristic (ROC) graph shown in Fig. 7 is a two-dimensional graph in which true positive rate versus false positive rate is plotted on the picture. The zoom in (+) version of the figure was used to view the saturation region more clearly. This graph described relative tradeoffs between true positives and false positives of eleven classifiers labeled 1 through 11 which actually represent অ to ঐ as mentioned in Table 2. In our ROC graph, it is seen that there is no point at the lower right triangle. It proves that this ANN classifier did a good job because any classifier that appears in the lower right triangle performs worse than random guessing (Flach and Wu 2005). In this case all eleven classes are in the upper triangular region.

**Fig. 8:**
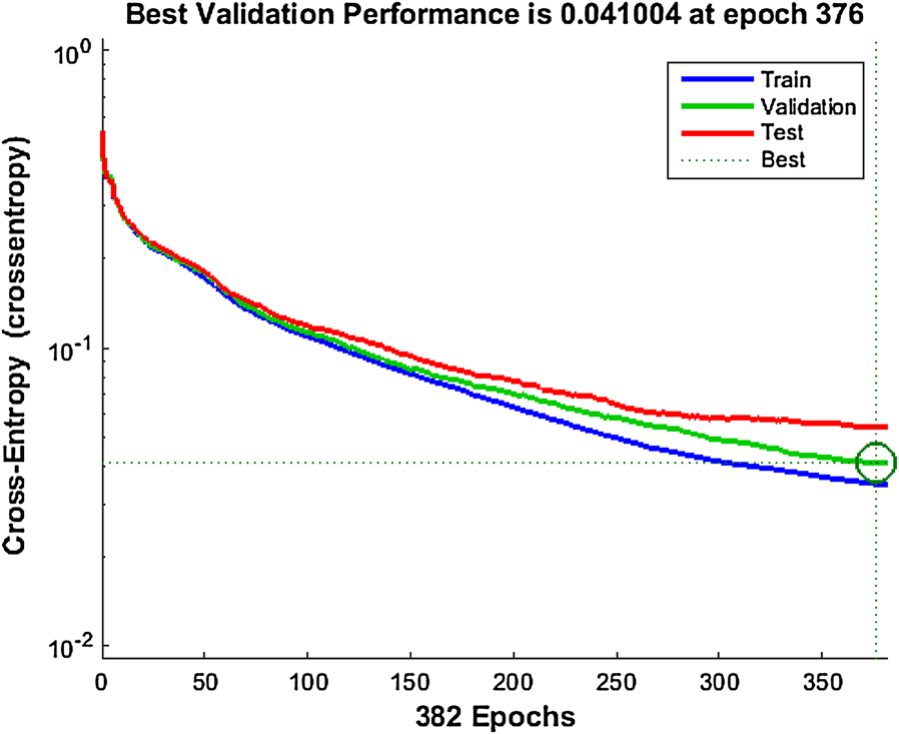
The performance *curve* showing the train, validation and test as well as best performance of the system

From Fig. 8 it is clear that the best validation occurred among the 376 epochs and the validation performance is 0.041004. After that, the validation performance became flat though the training performance is still going up (entropy value going down). The designed system chooses the results from 376 epochs to prevent overfitting in this particular case.

## Conclusions

This paper presents a novel approach to classify Bangla vowels. This approach used the facial muscle contraction using non-invasive sEMG signal. The present study extracted features from different domains and applied a state-of-the-art feature selection method called mRMR to select prominent feature subset. Finally, ANN is utilized to classify different vowels and achieved an overall classification accuracy of 82.3 % with only 12 features. The accuracy can be increased by adding more features. However, it is our intention to keep the number of features small that provide better classification accuracy. The methodology developed in this paper is not only useful in Bangla vowels classification but also useful in many biomedical research areas such as EEG seizure detection, brain-computer interface (BCI) etc.

A limitation of the present study is the number of subjects. Only 8 subjects were used in this study that may hampers on the accuracy rate. However, the aim of this work does not propose a final system, but to explore the possibility of developing such system. This novel work can be extended in a number of ways. The experiment needs to be done on voiceless people to validate the proposed method. The performance of the other neural networks like HMM, SVM with different kernels are needed to investigate for better accuracy. Our near future research will solve the present constraints and explore and extend the present methodology. Finally, it can be said that this novel method for Bangla vowels classification will help the voiceless people who can’t produce sound but can move their facial muscles just as the normal people.
